# Review of the MMSE as a Pre‐Screening Tool for Dementia in Clinical Trials

**DOI:** 10.1002/alz.090910

**Published:** 2025-01-03

**Authors:** Pamela Voccia, Noel Fundora, Cyntauria Jones, Sheniba Pennyfeather Thomas, Andrew Boateng‐Poku, Olumide Otekalu

**Affiliations:** ^1^ Alcanza Clinical Research, Clermont, FL USA; ^2^ Vertex Clinical Research, Clermont, FL USA; ^3^ NeuroStudies, Decatur, GA USA

## Abstract

**Background:**

The Mini Mental State Exam (MMSE) is a widely used prescreening tool in dementia research.

**Method:**

Psychometric Raters from two sites within the Alcanza Clinical Research network collected MMSE data from 85 potential subjects who presented for memory trials. Subjects were later screened for Alzheimer’s Dementia studies, and the results from subsequent administrations of the Clinical Dementia Rating scale were available for 53 of those subjects.

**Result:**

All 85 prescreening subjects endorsed memory concerns as the primary complaint. However, 56% (n = 48) scored within the Normal Cognition range (25‐30) on the MMSE.

Comparisons were made between some of the MMSE total scores and the calculated Global scores on subsequent administrations of the Clinical Dementia Rating scale (n = 53). The Clinical Dementia Rating Scale (CDR) is a semi‐structured clinical interview involving self‐report of memory, caregiver reports and clinician observations of cognition. The CDR is widely considered to be a reliable measure of the presence of symptoms related to dementia.

The range of the total MMSE scores for 53 subjects later evaluated with the CDR was between 18 and 28. Of those, all 53 (100%) were revealed to have symptoms of dementia based on CDR scores (CDR 0.5‐2), even though 33% of those patients had scored within the Normal range on the MMSE (25‐30; N = 18).

The average MMSE score for subjects falling within the Very Mild Dementia range (CDR = 0.5) was 23.9 with a standard deviation of 2.5 (n = 41). Of the subjects falling within this range, 41% scored within the Normal range on the MMSE (MMSE = 25‐28).

The average MMSE score for subjects falling within the Mild to Moderate Dementia range (CDR 1 or 2) was 21.2, with a standard deviation of 2.9 (n = 12). Of the subjects falling within this range, 1 patient (8%) scored within the Normal Range on the MMSE (MMSE = 26).

**Conclusion:**

Based on this limited analysis, it appears that using the MMSE alone as a screener for dementia may result in missing up to 33% of symptomatic patients. Within these sample groups, the initial self‐endorsement of memory concerns was a more reliable predictor of CDR outcomes than the MMSE.

